# Proteolytic Activation of Plant Membrane-Bound Transcription Factors

**DOI:** 10.3389/fpls.2022.927746

**Published:** 2022-06-14

**Authors:** Jonas De Backer, Frank Van Breusegem, Inge De Clercq

**Affiliations:** ^1^Department of Plant Biotechnology and Bioinformatics, Ghent University, Ghent, Belgium; ^2^Vlaams Instituut voor Biotechnologie (VIB)-Center for Plant Systems Biology, Ghent, Belgium

**Keywords:** membrane-bound transcription factors, proteolytic activation, regulated intramembrane proteolysis, intracellular signaling, stress response, *Arabidopsis thaliana*

## Abstract

Due to the presence of a transmembrane domain, the subcellular mobility plan of membrane-bound or membrane-tethered transcription factors (MB-TFs) differs from that of their cytosolic counterparts. The MB-TFs are mostly locked in (sub)cellular membranes, until they are released by a proteolytic cleavage event or when the transmembrane domain (TMD) is omitted from the transcript due to alternative splicing. Here, we review the current knowledge on the proteolytic activation mechanisms of MB-TFs in plants, with a particular focus on regulated intramembrane proteolysis (RIP), and discuss the analogy with the proteolytic cleavage of MB-TFs in animal systems. We present a comprehensive inventory of all known and predicted MB-TFs in the model plant *Arabidopsis thaliana* and examine their experimentally determined or anticipated subcellular localizations and membrane topologies. We predict proteolytically activated MB-TFs by the mapping of protease recognition sequences and structural features that facilitate RIP in and around the TMD, based on data from metazoan intramembrane proteases. Finally, the MB-TF functions in plant responses to environmental stresses and in plant development are considered and novel functions for still uncharacterized MB-TFs are forecasted by means of a regulatory network-based approach.

## Introduction

Membrane-bound TFs (MB-TFs) are TFs with at least one transmembrane domain (TMD) and they are present in all kingdoms, including viruses (Zupicich et al., 2001). Although TFs anchored to the membrane via lipid modifications (Eisenhaber et al., 2011; Duan et al., 2017) are sometimes classified as MB-TFs as well (Liu et al., 2018), we will use the most ‘stringent’ definition, namely that MB-TFs are “proteins that contain both a (predicted) TMD and a transcription factor family domain (TFFD)” (Kim et al., 2010; Yao et al., 2017). By their attachment to (sub)cellular membranes, MB-TFs are generally assumed to reside outside the nucleus in a dormant state until their release from the membrane in response to an intra- or extra-cellular trigger. Currently two mechanisms are known to reroute MB-TFs to the nucleus, either at the posttranscriptional level, by the generation of an alternative TMD-lacking transcript or by a proteolytic cleavage releasing the TF from the TMD (Kim et al., 2010). During the proteolytic activation, the MB-TF protein is cleaved between the TFFD and the TMD, thereby liberating the active TF from the membrane and enabling relocation to the nucleus because of the occurrence of a nuclear localization signal (NLS) in the remaining TF part. When the TMD is present at either the C- or N-terminus, a single proteolytic event is sufficient for release (Kim et al., 2010). The best-known mechanism is cleavage inside or proximal to the TMD by an intramembrane protease, also referred to as regulated intramembrane proteolysis (RIP) (Liu et al., 2018; Ye, 2020). Currently, one mechanism described thus far for the RIP activation of MB-TFs is conserved in plant, animal and yeast systems and mediated by a set of two intramembrane metalloproteases that release BASIC LEUCINE ZIPPER (bZIP) TFs from the endoplasmic reticulum (ER) during the unfolded protein response (Ye et al., 2000a; Stirling and O’Hare, 2006; Liu et al., 2007; Tajima et al., 2008). In metazoan and yeast systems, alternative release mechanisms have been described including auto-proteolytic activation, in which the proteolytic activity occurs in the MB-TF itself, as is the case for the vertebrate MYELIN REGULATORY FACTOR that is required for ER homeostasis maintenance (Bujalka et al., 2013; Milan et al., 2020). A related, but protease-independent, release mechanism translocates vertebrate epidermal growth factors upon hormone recognition to the nucleus by budding from the Golgi membranes via coat protein complex I-coated vesicles (Sigismund et al., 2008). In another mechanism, referred to as ubiquitin/proteasome-dependent processing (RUP), the ER luminal and transmembrane regions are degraded upon ubiquitination, leading to the release and nuclear translocation of the cytosolic MB-TF segment (Hoppe et al., 2000).

Recently, a comprehensive computational inventory of plant MB-TFs was generated by integrating the Plant Transcription Factor DataBase v4.0 with seven membrane topology predictors, resulting in 64 high-confidence *Arabidopsis* MB-TFs, further referred to as atMB-TFs (Yao et al., 2017). The atMB-TFs are found in 24 different TF families with the highest representation in the *Arabidopsis* NO APICAL MERISTEM/*ARABIDOPSIS THALIANA* ACTIVATING FACTOR/CUP-SHAPED COTYLEDON (NAC) and bZIP families, with 17 and 5 atMB-TFs, respectively. Here, we built on this inventory of atMB-TFs, assessed their subcellular localization and membrane topology, and identified novel potential cleavage events and structural features for proteolytic release based on *in silico* analyses. In this review, we also provide a thorough summary of the current knowledge on the proteolytic release mechanisms, the cellular and environmental MB-TF-activating triggers, and the MB-TFs involvement in plant responses to stresses and in plant development.

## MB-TF Activation Mechanisms

### Alternative Transcription

Posttranscriptional activation of MB-TFs involves the generation of a variant transcript isoform that no longer contains the TMD-encoding sequence. In plants, thus far, only two MB-TFs have been proven to be activated by alternative transcription: *Arabidopsis* bZIP60 and NAC with transmembrane motif 1-like 5 (NTL5) (Nagashima et al., 2011; Li et al., 2014). The bZIP60 transcript is spliced by the INOSITOL REQUIRING 1 (IRE1) endoribonuclease/kinase. Two IRE1 isoforms (IREa and IREb) are localized to the ER. Upon ER stress, provoked, for instance, by heat stress, dithiothreitol or tunicamycin treatment, with an unfolded protein response (UPR) as a consequence, they form homodimers that trigger their autoactivation, and consequently, the binding and splicing of an alternative 23-nucleotide intron that precedes the bZIP60 TMD (Deng et al., 2011; Nagashima et al., 2011). This IRE1-dependent alternative splicing causes a frameshift, resulting in a premature stop codon that excludes the TMD-encoding sequence from the mature transcript. This IRE1-dependent bZIP60 activation mechanism is similar to the previously discovered activation mechanism of the mammalian X-BOX BINDING PROTEIN 1 (XBP1) and the yeast HOMOLOGOUS TO ATF/CREB 1 (HAC1) bZIP TFs, hinting at the conservation of the UPR activation in eukaryotes. (Yoshida et al., 2001; Calfon et al., 2002; Cairrão et al., 2022). For NTL5, intron retention, leading to a premature TMD-preceding stop codon, was based on a single-nucleotide polymorphism (SNP) in the third intron of the *NTL5* gene in the Columbia-0 accession of *Arabidopsis*, leading to a permanent nuclear localization in this accession. In most of the other accessions, NTL5 is stored in the ER in a dormant state, until it is activated by still unidentified mechanisms in response to abscisic acid (ABA) stimulation (Li et al., 2014). This SNP-dependent activation caused by a mutation in the genome instead of an mRNA modification caused by the plant’s splicing machinery is designated differential (instead of alternative) splicing.

MB-TFs potentially activated by alternative splicing can be detected by searching for transcript variants in RNA-sequencing data (RNA-seq). Genome-wide analysis revealed that 18 (∼ 30%) of the 64 atMB-TF genes express annotated TMD-lacking alternative transcripts (Yao et al., 2017; Table 1). Remarkably, in the bZIP and NAC (or NAC with transmembrane motif-like, NTL) families, which represent the largest MB-TFs subfamilies, no transcripts without the predicted TMD were identified, except for ANAC050. This NAC TF is one of the two special cases, in which the TMD overlaps with its TFFD, hence the TMD could only be removed by alternative splicing. However, transcript variants can be missed from RNA-seq data, because particular alternative splicing events are often specific and depend on certain stimuli; for instance, the bZIP60 alternative transcript had only been detected under ER stress conditions (Nagashima et al., 2011). Of the 18 atMB-TF TMD-lacking transcript variants identified in RNA-seq data, 15 lost their TMD due to alternative splicing resulting in exon skipping. An example is the LSD ONE LIKE 1 (LOL1) zinc finger TF involved in the regulation of oxidative stress-induced cell death during the hypersensitive response (Epple et al., 2003). LOL1 has, according to RNA-seq data, seven different transcripts, of which only two contain a predicted N-terminal TMD (Epple et al., 2003). Thus far, LOL1 functional studies had been based only on a coding sequence without the predicted TMD. Thus, the existence of a TMD-containing protein isoform and its activation mechanism remain subjects for further study. For ZINC FINGER NUCLEASE 2 (ZNF2), an alternative transcript leads to a premature stop codon, similarly as for bZIP60, whereas for AINTEGUMENTA-LIKE 6 (AIL6) and LONESOME HIGHWAY LIKE 2 (LHL2), an N-terminal TMD-lacking alternative transcript hints at alternative splicing with a new translation initiation site as a result (Yao et al., 2017). Theoretically, activation of an MB-TF by alternative splicing does not rule out proteolytic cleavage, but activation by these two mechanisms in parallel has not been reported yet.

**TABLE 1:** Overview of protein domain organization, membrane topology, subcellular localization, structural features indicative for regulated intramembrane proteolysis (RIP) and alternative transcripts of membrane-bound TFs in *Arabidopsis* (atMB-TFs).

Protein name	TMD position relative to the TFFD	TMD score prediction^a^	Membrane topology^b^	Position^c^ (AA) of		Subcellular localization^d^	Postranslational modifications^e^	Helix-breaking residues in the TMD^f^	No. positively charged AA (K,R) in the^g^		No. alternative transcripts resulting in TMD loss Yao et al., 2017
				TFFD	TMD	Predicted| | experimental			TMD	20-AA TM-flanking region	
AIL6	N	0.702	I	389-440	301-321	mt, nuc**| | mt**	ph(4)		0	K(3) R(1)	2
ANAC028	C	0.570		6-143	609-630	Nuc	nt(1)*		K(2)	K(5) R(1)	
AT2G13960	N	0.582	II	40-86	9-29	sec, nuc, PM		[GxxN]	0	K(4)*	
AT2G29660	N	0.477	I	126-149	9-29	sec, mt, nuc	ph(1)*		R(1)	K(6) R(4)*	
AT3G04930	C	0.486	II	137-235	372-395	nuc**| | PM**	ph(5)*		0	K(1)	1
AT5G25475	N	0.606	I	78-155	52-75	Mt		[PxxN]	K(1)	K(1)R(5)*	2
AT5G63280	C	0.836	II	105-128	223-246	sec, nuc	nt(1) ng(1)* ac(1) ro(1)		0	K(4) R(1)*	
bHLH035	C	0.581	II	58-101	203-224	Nuc	ph(1)*		0	K(2)	3
		0.360	II		190-213				K(1)	K(2) R(1)	
		0.871	I		214-245			[PN]	R(1)		
bHLH115	C	0.363		136-182	190-211	Nuc			K(5)	K(1) R(2)	
bHLH131	N	0.463		1351-1397	1242-1262	nuc, sec**| | pl and mt**	ph(1)		0	K(2)	2
		0.341			1277-1297				0		
bZIP7	N	0.662		197-254	38-59	Nuc	ph(3)* ng(1)		K(1)	0	3
bZIP17	C	0.798	I	226-287	364-387	nuc**| |** ER, PM, nuc [1]	ph(1)	[GA]	K(1)	K(7)*	
bZIP28	C	0.848	I	192-237	321-344	nuc, sec, ER**| |** ER, PM, nuc [2]			K(1)	K(7) R(1)*	
bZIP49	C	0.755	I	172-219	286-309	Nuc		[GA]	K(1)	K(6) R(1)*	
bZIP60	C	0.693	II	141-183	217-240	Nuc			0	K(2) R(1)	
CAMTA1	C	0.380	I	81-188	192-211	Nuc	ph(10)* na(1) nt(1)		R(1)	K(1) R(3)	
CAMTA5	C	0.683		30-146	579-600	nuc**| | Golgi(4), PM, nuc**	ph(3)* sm(1)		0	K(4)	1
FRF3	C	0.424		25-110	115-136	mt, cyt			R(1)	K(2) R(1)	1
GPL2	C	0.786		61-155	328-348	Nuc	ph(4)*	[PxxP]	0	0	
HHO5	N	0.567		218-272	64-85	Nuc	ph(1)		R(1)	K(3) R(1)	3
LD	C	0.496		66-122	222-243	Nuc	ph(2)* ub(1)		K(1)	K(2) R(1)	
LHL2	N	0.537		529-566	123-144	nuc**| |** nuc [3]			0	K(1) R(4)	2
LOL1	N	0.488	II	70-171	24-47	sec, nuc			0	K(3) R(1)	
MAMYB	N	0.770	I	159-193	35-55	nuc**| | ER(2), Golgi(4)**,** PM(3),** nuc [4]	ph(14)* na(1)		0	K(1)	
		0.575	II		61-84			[PxxP]	K(1)		
NFXL2	C	0.462	II	247-452	840-863	Nuc	ph(1)*		K(1)	K(2) R(2)	
NGAL2	C	0.469	II	29-142	198-221	Nuc			0	K(2)	2
NLP3	N	0.550	I	495-546	41-64	mt, nuc			0	0	
NOK	N	0.345		57-155	30-51	nuc**| |** nuc [5]	nt(1)		K(1) R(1)	K(1) R(1)	
NTL1	C	0.792	I	10-135	497-520	nuc**| |** ER, PM, nuc [6]		[GA]	0	K(7) R(1)*	
NTL2	C	0.760	II	24-151	605-625	nuc**| |** ER, PM, nuc [6]			0	K(5) R(1)*	
NTL3	C	0.718	II	17-143	535-555	nuc**| |** ER, PM, nuc [6]		[GA]	K(1)	K(2) R^2^	
NTL4	C	0.714	II	9-136	522-545	nuc, PM**| |** ER, PM, nuc [6]			K(1)	K(1)	
NTL5	C	0.528	II	15-140	316-334	nuc**| |** ER, PM, nuc [6]			R(1)	K(1) R(1)	
NTL6	C	0.523	II	13-141	442-462	nuc**| |** ER, PM, nuc [6]	ph(9)*		R(1)	K(3) R(1)	
NTL7	C	0.856	II	17-143	525-548	nuc, cyt**| |** ER, PM, nuc [6]	ph(1)*	[GA]	R(1)	K(3) R(2)	
NTL8	C	0.759	II	14-140	312-332	nuc, PM**| |** ER**, PM,** nuc [6]			0	K(4) R(2)	
NTL9	C	0.528	II	9-135	488-511	nuc, Golgi**| |** ER, PM, nuc [6]			K(1) R(1)	K(2) R(5)*	
NTL10	C	0.773	II	5-138	410-428	nuc**| |** ER, PM, nuc [6]	ph(3)*		0	K(2)	
NTL11	C	0.571	II	9-136	541-564	nuc**| |** ER, PM, nuc [6]	ph(2)*		K(1) R(1)	K(1)	
NTL13	C	0.480	II	22-147	319-339	nuc**| |** ER, PM, nuc [6]			0	K(2) R(1)	
NTM1	C	0.432	II	6-136	445-468	nuc**| |** ER, PM, nuc, **cytoskeleton** [6]	ph(4)*		K(2) R(2)	K(4) R(2)	
OBP3	N	0.595		118-177	42-62	mt, nuc	ph(2)*		0	0	5
RLT2	C	0.554		18-74	518-539	nuc**| | Golgi**	ph(24)* ac(1)*		R(1)	K(4)	3
SCP	N	0.550	II	36-136	14-37	sec, nuc**| |** nuc [7]		[GA]	R(1) K(1)	K(1) R(3)	
SPL1	C	0.641	II	105-182	835-858	nuc**| |** ER, PM, nuc [8]	ph(3)*		R(1)	K(2) R(2)	
SPL7	C	0.627	II	137-213	762-782	nuc**| |** nuc [9]	ph(1)*		0	K(2) R(3)	
SPL12	C	0.641	I	126-203	881-904	nuc**| |** ER, PM, nuc [8]	ph(10)*		R(1)	K(3) R(1)	
SPL14	C	0.614	II	119-196	995-1018	nuc**| |** ER, **PM**, nuc [8]	ph(3)*		0	K(1) R(3)	
SPL16	C	0.614	II	82-158	978-1001	nuc**| | PM**			0	K(2) R(3)	
SRS8	C	0.375	II	46-140	144-164	Nuc	ph(1)		0	K(1) R(1)	3
WIP4	N	0.468		256-362	42-63	nuc, sec			0	K(1) R(1)	
ZFN2	C	0.720		45-334	444-465	Nuc	ph(8)* na(1) nt(1) my(1) ub(1)		K(1)	0	2

*cyt, cytosol; pl, plastid; nuc, nucleus; sec, secretory pathway; PM, plasma membrane; mt, mitochondria; ph, phosphorylation; na, N-terminal acetylation; nt, N-terminus proteolysis; ng, N-glycosylation; ac, lysine acetylation; ro, reversible cysteine oxidation; sm, lysine SUMOylation; ub, lysine ubiquitination; my, myristolysation.*

*^a^Mean hydrophobicity within the TMD obtained from Aramemnon (Schwacke et al., 2003), with mean hydrophobicity value > 0.68 representing a high score and < 0.42 representing a low score.*

*^b^Membrane topology prediction obtained from TMHMM (Krogh et al., 2001), with type-I and type-II referring to a membrane-bound protein with the C-terminus and the N-terminus in the cytosol, respectively.*

*^c^Position of the TFFD and TMD obtained from the PlnTFDB v5.0 database (Riaño-Pachón et al., 2007) and TMHMM (Krogh et al., 2001) or from Aramemnon (Schwacke et al., 2003), respectively.*

*^d^The predicted subcellular localization obtained from SeqNLS (Lin et al., 2012), SignalP 6.0 (Teufel et al., 2022), DeepSig (Savojardo et al., 2018), and Aramemnon (Schwacke et al., 2003) is presented in Supplementary Table 2; the experimentally determined subcellular localization based on fluorescent protein fusion (regular text), mass spectrometry analysis of subcellular fractions (bold) and both methods (underlined) was obtained from SUBA4 [2] Liu et al. (2007); [7]Oh et al. (2010); [1] Liu et al. (2008); [4] Slabaugh et al. (2011); [3] Ohashi-Ito et al. (2013); [6] Liang et al. (2015); [8] Chao et al. (2017), Hooper et al. (2017); [9] Ramamurthy et al. (2018); [5] Hong et al. (2021).*

^e^Between parentheses, the number of modifications; *indicates that at least one of them is present in between the TMD and TFFD, with the amino acid positions indicated in Supplementary Table 3.

*^f^Amino acid position in the TMD are presented in Supplementary Table 4.*

^g^Number of lysines (K) and number of arginine (R) are indicated between parentheses; *significantly enriched compared to the Arabidopsis proteome or compared to the TMD regions encompassing the 5–, 10–, 15– or 20-amino-acid flanking regions of the Arabidopsis membrane-bound proteome (Bonferroni-corrected hypergeometric P values < 0.05)., with corresponding P values presented in Supplementary Table 7.

### Proteolytic Cleavage

The *Arabidopsis* bZIP17 and bZIP28 are the only known plant MB-TFs, for which the proteolytic release mechanisms are well characterized and the responsible proteases have been discovered (Liu et al., 2007; Tajima et al., 2008). For years, the SITE-1 PROTEASE (S1P) metalloprotease has been assumed to be responsible for cleavage of bZIP17 and bZIP28 at their > 300 amino acid-long tail in the ER lumen, resulting in their translocation to the Golgi whereas S2P to be responsible for the subsequent release of both TFs from the Golgi membranes, likewise to the well-described activation of STEROL REGULATORY ELEMENT-BINDING PROTEIN (SREBP) bZIP TFs in mammalian systems (Ye et al., 2000a; Stirling and O’Hare, 2006). This hypothesis was based on mutation of the S1P canonical sequence in bZIP17 and bZIP28 that resulted in the loss of the bZIP17 and bZIP28 target genes activation as well as in a deficient UPR (Sun et al., 2013, 2015). Cleavage patterns of bZIP17 in an *s1p* mutant background showed that bZIP17 is cleaved by S1P, but the second cleavage, anticipated to be carried out by S2P, has not been explicitly demonstrated. However, analysis of bZIP28 cleavage patterns in the *s1p* and *s2p* mutant backgrounds, confirmed cleavage only by S2P and not by S1P, indicating that the first cleavage event of bZIP28 is done by another, still unknown protease (Iwata et al., 2017). This implies that, in addition to S1P and S2P, in plants, (an)other protease(s) are involved in the proteolytic release of these bZIP TFs (Figure 2).

**FIGURE 1:**
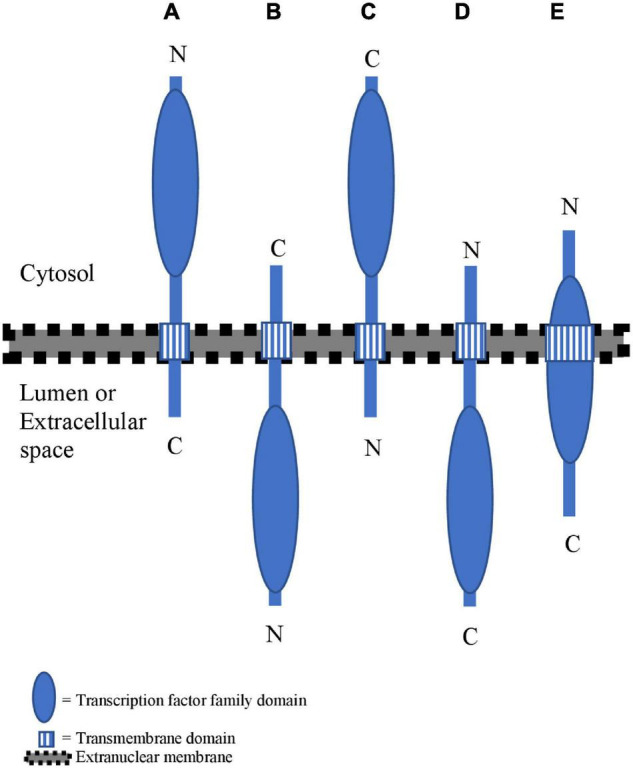
Overview of different membrane topologies for membrane-bound TFs (MB-TFs). **(A)** MB-TFs with the transmembrane domain (TMD) at the C-terminal side of the transcription factor family domain (TFFD) and a type-II membrane topology. **(B)** MB-TFs with the TMD at the C-terminal side of TFFD and a type-I membrane topology. **(C)** MB-TFs with the TMD at the N-terminal side of TFFD and a type-I membrane topology. **(D)** MB- TFs with the TMD at the N-terminal side of TFFD and a type-II membrane topology. **(E)** MB-TFs with the TMD overlapping with the TFFD.

**FIGURE 2:**
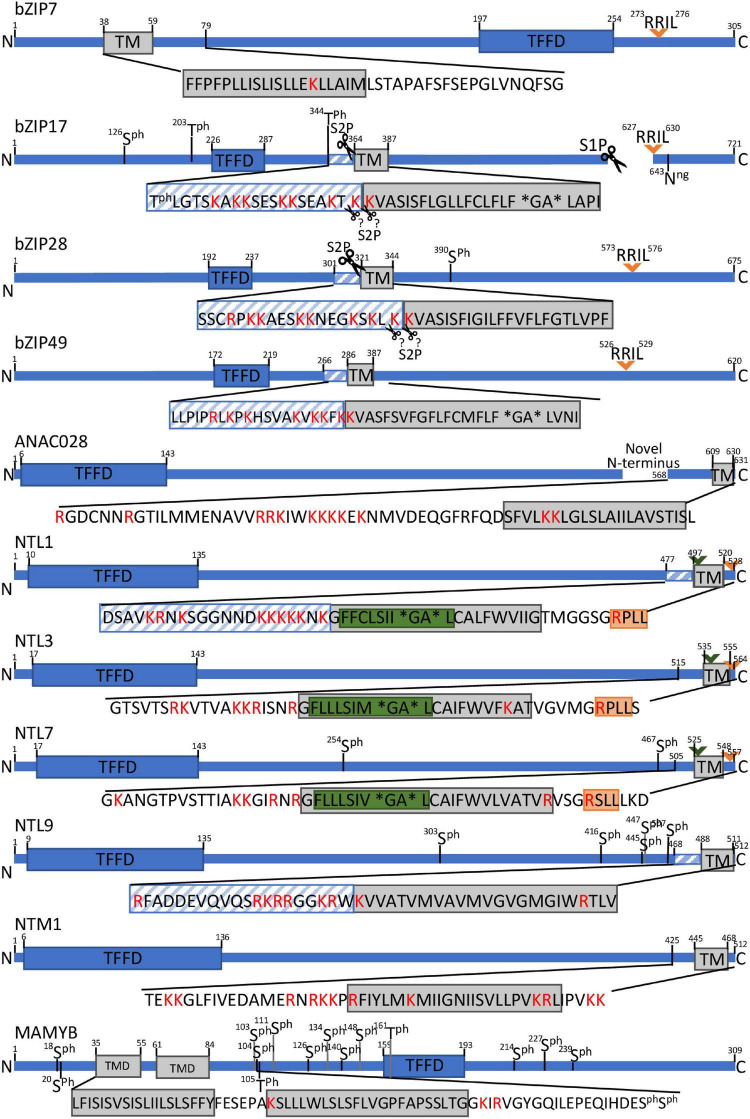
Schematic presentation of protease recognition motifs and structural features indicative of RIP activation for a selection of MB-TFs. Predicted rhomboid recognition sites (LxLSIxGA) are indicated in green and (predicted) SIP recognition sites (Rx[LIT][KL]) in orange. Diagonal blue stripes mark TMD-neighboring regions that are significantly (corrected *P* value < 0.05) enriched for positive amino acids (R and K. lysine and arginine) and scissors indicate known or predicted cleavage sites. Asterisks specify helix-breaking residues. Ph. phosphorylated amino acid.

Furthermore, members of the NAC family have been studied for proteolytic activation. NAC WITH TRANSMEMBRANE MOTIF1 (NTM1) exchanges an ER to a nuclear localization pattern during cell division induced by kinetin or cytokinin (Kim et al., 2006; Kim and Park, 2007). Accordingly, a shortened protein isoform was detected by protein immunoblot analysis, with a size corresponding to that of a truncated isoform without TMD. Interestingly, pretreatment with the calpain protease inhibitor N-acetyl-leucinyl-leucinyl-norleucinal (ALLN) attenuated the nuclear relocalization and the truncated isoform detection, hinting at cleavage by a cytosolic non-membranous protease (Kim et al., 2006). In mammals, only the Nuclear Respiratory Factor 1 (Nrf1) MB-TF is known to be activated by a cytosolic protease, the cytosolic aspartic protease DNA damage-inducible 1 homolog 2 (DDI2), but this occurs after retrotranslocation of the TFFD from the luminal to the cytosolic side of the ER membrane through ER-associated degradation (ERAD) complex (Chen et al., 2022).

**FIGURE 3:**
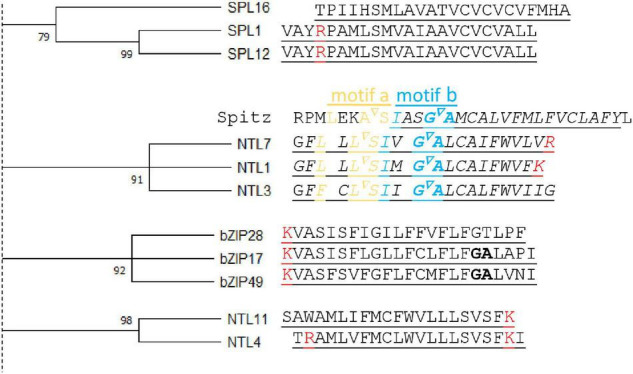
Transmembrane domain similarities of atMB-TFs. Left, A bootstrap (1,000 replicates) consensus tree was constructed from all atMB-TF transmembrane domains (TMDs) (Tamura et al., 2021) (for the complete tree, see Supplementary Figure 1). Here, only clusters with bootstrap values higher than 70 are displayed. Right, Alignment of the TMD amino acid sequences with positive amino acids (lysine and arginine, red) and helix-breaking residues (bold) indicated. For NTL7, the alignment is presented with the *Drosophila melanogaster* Rhomboid-1 recognition (motifs a and b) and cleavage (inverted triangle) sites from the Spitz substrate TMD region.

NTL1, NTL3, and NTL7 have been proposed to be activated by a comparable mechanism due to the high sequence similarity in their C-terminal region that harbors the (predicted) TMD (Figure 3). NTL1 and NTL7 are localized to the ER and function under conditions that perturb the mitochondrial reactive oxygen species (ROS)/redox status or induce mitochondrial dysfunction, resulting in their nuclear translocation and activation of genes involved in oxidative stress responses (De Clercq et al., 2013; Ng et al., 2013). How perturbed mitochondria signal to the ER to trigger and release these TFs is still not understood, but clearly hint at a proteolytic event, because the mitochondrial stress-induced expression of the NTL1/3/7 target genes was attenuated by pretreatment with the serine protease inhibitor N-p-Tosyl-L-phenylalanine chloromethyl ketone (TPCK). Moreover, the TMD of NTL7 contains a conserved dual cleavage site of the well-studied *Drosophila melanogaster* Rhomboid 1 (Rho-1) at the presumed cytosolic side, indicating cleavage by a yet unidentified rhomboid protease (Figure 2).

## Membrane Targeting and Topology of atMB-TFs

The subcellular localization of MB-TFs is predominantly defined by their TMD, but once the TMD is removed, the TF is directed to the nucleus by the presence of a NLS. We assessed the subcellular localization of the atMB-TFs by combining *in silico* predictions and experimental evidence based on fluorescence labeling and mass spectrometry analysis of purified subcellular compartments. According to the SeqNLS algorithm (Lin et al., 2012), 73% of the atMB-TFs contain a NLS (Table 1), but because SeqNLS was shown to predict only a NLS for 80% of the known nuclear proteins in a yeast training dataset (Lin et al., 2012), other atMB-TFs might have a still undiscovered NLS. In addition, a dual, nuclear and non-nuclear, localization for one and 15 (1.5 and 23.4%) and an exclusive non-nuclear localization for eight and three (12.5 and 4.7%) out of the 64 atMB-TFs were predicted by SUBA4 (Hooper et al., 2017) and Aramemnon (Schwacke et al., 2003), respectively (Table 1). The reason for this low number of dual and extranuclear predictions is that these programs mostly rely on targeting or signaling peptides and not on the TMD-based localization. Presumably, the TMD is sufficient for extranuclear targeting, because the TMD of NTL7 was shown to have a similar localization pattern as the full length protein (Ng et al., 2013). Moreover, proximity-specific ribosome profiling experiments revealed that ribosomes that translate membrane-anchored proteins, including MB-TFs, typically target and bind to the ER-localized translocons just before or after the TMD translation (Jan et al., 2014). However, how these proteins further traverse the secretory pathway to reach their final destination, and whether this is dictated by the TMD and/or by additional signal peptides, is still not clear (Yang et al., 1997).

Eleven atMB-TFs were experimentally identified in one or more isolated subcellular compartments, including the plasma membrane (AGAMOUS-LIKE 69, NTL8, SQUAMOSA PROMOTER BINDING PROTEIN-LIKE 14 [SPL14], SPL16 and a DUF573 family TF [AT3G04930]), the Golgi (RINGLET 2 [RLT2]), the ER, Golgi and plasma membrane (MEMBRANE ANCHORED MYB [MAMYB]), the mitochondria [AIL 6], the plastids and mitochondria (basic HELIX-LOOP-HELIX [bHLH131]), the cytoskeleton [NTL12], and the Golgi, plasma membrane and nucleus (CALMODULIN-BINDING TRANSCRIPTION ACTIVATOR 5 [CAMTA5]) (Hooper et al., 2017; Table 1). Surprisingly, with the exception of CAMTA5, none of the atMB-TFs were identified in isolated nuclei (Hooper et al., 2017). Possibly, these TFs might be present only in the nucleus when they are activated under certain conditions and, hence, escape detection in the nucleus under basal conditions. However, based on fluorescent protein tagging, both nuclear and membrane-bound localization patterns could be shown for 18 atMB-TFs (Liu et al., 2007, 2008; Oh et al., 2010; Slabaugh et al., 2011; Ohashi-Ito et al., 2013; Liang et al., 2015; Chao et al., 2017; Ramamurthy et al., 2018; Hong et al., 2021; Table 1).

To enable nuclear translocation after cleavage, the TFFD-containing protein part should principally reside on the cytosolic membrane side. The best-studied mechanism for proteolytic release of such MB-TFs is through a single proteolytic event inside or close to the TMD by an intramembrane protease (Liu et al., 2018; Ye, 2020). However, the TFFD may not always be present in the cytosol under basal conditions. For example, the TFFD of the mammalian NRF1 occurs in the ER lumen, but is retrotranslocation to the cytosolic side through the ERAD complex that ubiquitinates and shuttles ER proteins to the cytosol for their degradation by the proteasome. The cytosolic-oriented NRF1 TFFD is subsequently cleaved between the TMD and the TFFD by the cytosolic DDI2 protease to escape from proteasomal degradation and eventually translocate to the nucleus (Zhang et al., 2014; Chen et al., 2022). To assess the membrane organization of the atMB-TFs, we computationally evaluated the positions relative to the TFFD of the TMDs and their membrane topology. Similar to the study of Yao et al. (2017), the TFFD position within the MB-TF and the TMD position and orientation were retrieved from the PlnTFDB v5.0 (Riaño-Pachón et al., 2007) and the TMHMM predictor (Krogh et al., 2001) and Aramemnon tool that integrates 18 different TMD-predicting algorithms (Schwacke et al., 2003; Schwacke and Flügge, 2018), respectively (Table 1). Based on the TMD position, we distinguished three atMB-TF groups with the TMD (i) on the C-terminal side of the TFFD (60.3%) (Figures 1A,B), (ii) on the N-terminal side of the TFFD (23.8%) (Figures 1C,D), and (iii) overlapping with the TFFD (15.9%) (Figure 1E). Most atMB-TFs, including all NTLs and all bZIP MB-TFs, except bZIP7 belong to group (i), of which 59.5% and 21.5% are predicted to have a type-II and a type-I membrane topology, i.e., the N-terminal TFFD is present in the cytosol (Figure 1A) and the organelle lumen or cell exterior (Figure 1B), respectively, whereas for 19% no membrane topology was predicted. For MB-TFs with the TMD on the N-terminal side (group ii), only 26.6% are predicted to have a type-I orientation that would result in the C-terminal TFFD at the cytosolic side (Figure 1C) and 26.6% to have the opposite topology (Figure 1D), whereas for 46.6% the prediction was lacking. This resulted in 28 (54%) atMB-TFs with the TFFD predicted at the cytosolic side of the membrane, including all NTLs, except NTL1. However, for most MB-bZIPs that have a predicted membrane topology, the TFFD is predicted in the lumen. Nevertheless, the prediction of the membrane topology remains challenging and is based on homology to proteins with experimentally verified topologies (Käll et al., 2007). The TFFDs of bZIP17 and bZIP28 had been predicted to be on the luminal side of the ER and Golgi, respectively, but treatment of permeabilized cells with a non-specific protease revealed that the TFFDs of both TFs were accessible to protease digestion, thus residing on the cytosolic side (Gao et al., 2008). In addition to protease digestion assays, the predicted membrane topology of TMDs is sometimes experimentally validated by a self-assembling split fluorescent protein (FP) system, in which one half is targeted to one side of the TM protein and the other to the cytosol or organelle lumen (Bujalka et al., 2013). When the TF is present at the cytosolic side, the two GFP parts spontaneously assemble and a fluorescent signal is detected.

The presence of a predicted TMD within the TFFD (group iii) seems illogical and if it were a true TMD, activation through alternative splicing instead of proteolytic cleavage would be the only manner to ‘activate’ these TFs. Two of the 10 atMB-TFs with a TFFD-overlapping ‘hydrophobic peptide’, ANAC050 and S1Fa1, have a transcript isoform lacking it, hinting at activation of these MB-TFs by alternative splicing (Supplementary Table 1; Yao et al., 2017). These hydrophobic peptides in the TFFD might plausibly be important for DNA binding or for proper folding of the TFs instead of for membrane anchoring (Zhou et al., 1995; Ogata et al., 1996). atMT-TFs for which the TMD overlaps with the TFFD were therefore excluded for further *in silico* analysis of proteolytic activation mechanisms in this review.

## *In silico* Evidence for Proteolytic Activation of atMB-TFs

### Intramembrane Protease Recognition Sites in atMB-TFs

Before the identification of specific sequences and structural features indicative of intramembrane proteolysis, we carried out a comparative analysis of the transmembrane domains among the different atMB-TFs. Previously, the TMD of substrates of intramembrane proteases from the same family had been shown to be similar in their TMD amino acid sequence (Beel and Sanders, 2008; Strisovsky et al., 2009). We assessed whether similarities between TMDs could infer proteolytic activation of atMB-TFs. Therefore, we constructed a bootstrap consensus tree for all atMB-TF TMDs and identified five clusters with bootstrap values higher than 90 (Figure 3). The TMDs of bZIP17 and bZIP28, both known to be activated through S2P cleavage, and bZIP49, predicted to be activated by S2P (Tajima et al., 2008; Iwata et al., 2017), but not bZIP60 that is regulated by alternative splicing (Nagashima et al., 2011), cluster together in our analysis, hinting at the competence to differentiate between RIP and alternative splicing regulation by means of TMD sequence similarity. Moreover, the ER membrane-bound NTL7, NTL1, and NTL3 with partially redundant functions in mitochondrial retrograde signaling have highly conserved TMDs as well (De Clercq et al., 2013; Ng et al., 2013). As mentioned, NTL7 is possibly activated by RIP (Ng et al., 2013) and interestingly, we also found the Rho-1 recognition site in NTL1 and NTL3 (see below), indicating that these TFs are probably also activated through RIP and, presumably, by (the same or different) rhomboid proteases (Figure 3). NTL4 and NTL11, which are localized to the ER as well and are, among other functions in abiotic stress responses, also involved in mitochondrial retrograde signaling (Morishita et al., 2009; Yabuta et al., 2011; Lee et al., 2012, 2014; De Clercq et al., 2013; Shih et al., 2014; Gladman et al., 2016; Van Aken et al., 2016), have a TMD dissimilar from that of NTL1/3/7 (Figure 3). Although the proteolytic activation of NTL4 and NTL11 has not been evidenced, NTL11 contains a phosphorylation site that is essential for production of a nuclear isoform, indicating activation at the posttranslational level and through proteolysis (Tang et al., 2016). NTL4 and NTL11 have no Rho-1 recognition site and are, therefore, likely cleaved by other proteases.

Next, we analyzed all atMB-TF protein sequences for previously identified recognition sequences for intramembrane proteases, because, thus far, no MB-TF is known to be released from the membranes by a soluble protease. Naturally, the release by a soluble protease cleaving the MB-TF in its cytosolic part cannot be excluded, as this activation means has been proposed for NTM1. Cleavage by a cytosolic calpain has been put forward, after immunoblot analysis had revealed that the calpain inhibitor ALLN altered the NTM1 cleavage pattern (Kim et al., 2006).

In plants, three intramembrane protease families are distinguished: intramembrane metalloproteases, rhomboid proteases, and intramembrane aspartyl proteases. Currently only one plant intramembrane protease recognition sequence has been identified, namely the S1P recognition site “RRIL”. This sequence was deduced from the consensus “Rx[LIT][KL]” (with x any amino acid) of the mammalian S1P substrates and in *Arabidopsis* first characterized in the bZIP28 (Denard et al., 2011; Iwata et al., 2017; Ye, 2020). In contrast to that of S1P, the S2P recognition site still awaits identification, both in mammals and in plants but is known to depend on structural elements rather than on sequence motifs in the TMD (see below). The S1P recognition sequence “RRIL” is present in four out of the 57 atMB-TFs, namely the previously reported bZIP17, bZIP28, and bZIP49 (Liu et al., 2007, 2008; Sun et al., 2015), and the additionally found hit in bZIP7 (Supplementary Table 1). Similarly as the mammalian S1P substrates that are cleaved inside the ER lumen, RRIL is present in the luminal tail of bZIP17, bZIP 28, and bZIP49, but its location (cytosolic or luminal) for bZIP7 remains unclear due to the lack of confident orientation prediction (Table 1). To discover whether other atMB-TFs also contain a potential S1P recognition site, we mapped the more degenerate consensus S1P recognition motif Rx[LIT][KL] (Denard et al., 2011). This motif was found in 38 atMB-TFs, corresponding to a 1.50-fold overrepresentation (67 vs. 45%; *P* value = 0.001, hypergeometric distribution) and a 1.33-fold enrichment (67% vs. 50%; *P* value = 0.02, hypergeometric distribution) when compared to the background *Arabidopsis* proteome (obtained via UniProt; Bateman et al., 2021) and all membrane-bound proteins in *Arabidopsis* (obtained via the *Arabidopsis* membrane protein library; Ward, 2001), respectively (Supplementary Table 4). As intramembrane proteases are generally expected to cleave in or close to the TMD, we restricted the search window to the TMD and the TMD region encompassing the 20-amino-acid flanking sequences. We identified Rx[LIT][KL] in seven atMB-TFs (NTL1, NTL3, NTL7, FAR1-RELATED SEQUENCES-RELATED FACTOR 3 [FRF3], CAMTA5, NIN-LIKE PROTEIN 3 [NLP3], and AT5G63280) and more specifically, in the 20-amino-acid surrounding, but not in the TMD, region. However, this approach did not result in a clear overrepresentation, based on the comparison to the 20-amino-acid region around the TMD of all membrane-bound proteins in *Arabidopsis* (12% versus 8%; *P* value = 0.3, hypergeometric distribution). These enrichment analyses indicate that this sequence is too small and degenerate and that additional sequence and/or structural features need to be taken into account to identify *bona fide* S1P targets. Another important criterium is the subcellular localization of the S1P cleavage, for several bZIP MB-TFs known to occur in the luminal tail. Based on the membrane topology predictions (Table 1), the Rx[LIT][KL] sequence was predicted, besides for bZIP17, bZIP28, and bZIP49, also on the luminal side for NTL3, NTL7, NPL3, and AT5G63280.

Thus far, for rhomboid proteases, only one potential recognition site has been identified in plants, of which the sequence was inferred from the *Drosophila* Rho-1 recognition site in the SPITZ substrate (Strisovsky et al., 2009). This sequence motif “LxxASIxxGA” includes two redundant cleavage sites: between alanine (A) and serine (S) and between glycine (G) and alanine (A). When the AS-encoding sequence is mutated, SPITZ is cleaved at the second, but less favored, cleavage site between G and A (Strisovsky et al., 2009). On the other hand, the “GA” sequence is also known as a structural feature for RIP (see below). A similar recognition sequence, “LxLSIxGA” had been identified in the TMD of *Arabidopsis* NTL7 (Ng et al., 2013). Although this predicted rhomboid recognition site still awaits validation in plants, pharmacological inhibition assays with the rhomboid inhibitor TPCK demonstrated attenuation of the NTL7 target gene promoter activation in response to mitochondrial stress (Ng et al., 2013). The “[LF]xLSIxGA” sequence also occurs in the closely related NTL1 and NTL3 and only in one additional protein in the *Arabidopsis* proteome, i.e., the tonoplast-located nitrate transporter NPF5.11 (He et al., 2017), corresponding to a 399-fold enrichment among the MB-TFs when compared to the background proteome (*P* value = 8e-166; hypergeometric distribution) (Supplementary Table 4). Other rhomboid recognition sequences, namely those of the bacterial proteases, AarA and GlpG/YqgP, and *Drosophila* Rho-1 and Human PARL, identified in the TatA, LacYTM2, Gurken/SPITZ, and PINK1, substrates, respectively, “[ILMF]xx[GAS][AHS][IMLF]” and “[ILMF]x[GAS][AHS][IMLF]”, could not be identified in or around the TMD of the atMB-TFs (Strisovsky et al., 2009; Deas et al., 2011) (Supplementary Table 4). Therefore, we searched the minimal consensus rhomboid recognition sequence conserved for both animal and bacterial rhomboids ([^WP][IMYFWLV][^WPD][^WF][AGCS][^P][FIMVACLTW]) (with ^ corresponding to all, but the amino acids indicated) (Strisovsky et al., 2009) and found it in the TMD and in the TMD region encompassing the 20-amino-acid flanking sequences of most (86 and 93%, respectively) atMB-TFs, demonstrating that this sequence is too degenerate to predict RIP in plants (Supplementary Table 4).

The third group of intramembrane proteases are the aspartyl proteases that encompass signal peptidases, presenilins, and γ-secretase. We did not carry out a sequence analysis, because no recognition sequences are well defined, the sequence conservation among substrates is minimal, and targeted substrate mutations are well tolerated. Thus, secondary and higher-order structures are more important for substrate recognition than sequence motifs (Beel and Sanders, 2008).

### Structural Features for Regulated Intramembrane Proteolysis

Substrate specificity for intramembrane proteolysis is, besides a protease recognition sequence, highly dependent on structural features in the TMD. Mutations resulting in conservative amino acid changes, such as replacement of a small hydrophobic amino acid with another small hydrophobic acid, have mostly no effect on the activation, whereas major changes, such as exchange of small hydrophobic amino acids by large ones, even when far from the cleavage site, often reduce or even completely abolish the proteolytic activation (Ye et al., 2000a,b; Strisovsky et al., 2009). Moreover, inversion of the TMD, removal of helix-breaking residues, or mutation of positively charged amino acids close to the TMD also reduced or even completely abolished cleavage, as reported for S1P substrates (Ye et al., 2000a,b; Beel and Sanders, 2008; Ye, 2020). Similarly, for rhomboid and aspartyl intramembrane protease substrates, which are cleaved inside the TMD, secondary protein structures, such as broken α-helixes or the α-helix and random coil interface, have been shown to be important for cleavage by Signal Peptide Peptidases, λ-secretase, and rhomboids (Beel and Sanders, 2008). Such secondary structures are considered essential for protease accessibility and binding to the recognition sequence because they weaken the protein structure within the membrane (Strisovsky et al., 2009).

We searched the atMB-TF TMDs for helix destabilization motifs based on the presence of helix-breaking residues, i.e., “NP” (Ye et al., 2000a), “NxxP” (Ye et al., 2000b), “PxxP” (Denard et al., 2011), and “GxxN” (Ye, 2020), found to be necessary for S1P-dependent RIP events in animal models, and “GA”, required for cleavage by animal and bacterial rhomboids (Hooper and Lendeckel, 2007). As the orientation of the protease relative to the substrate is not always determined in plants, the helix destabilization motifs were examined both in forward and reverse orientation. We identified one helix destabilization motif within the TMDs of 11 out of the 57 atMB-TFs, including GEBP-like protein 2 and MAMYB (“PxxP”), the Myb TF AT2G13960 (“GxxN”), bHLH035 (“PN”), the AP2/B3-like TF AT5G25475 (“PxxN”), NTL1, NTL3, NTL7, bZIP17, bZIP49, and SIDECAR POLLEN (SCP) (“GA”) (Table 1). These motifs are equally represented in TMDs of all *Arabidopsis* membrane-bound proteins and none of them appears significantly overrepresented in the atMB-TFs when compared to the *Arabidopsis* membrane-bound proteins (Supplementary Table 5). Interestingly, the “GA” motif is present in five atMB-TFs, NTL1/3/7 and bZIP17/49, known or predicted to be regulated by RIP.

Besides helix-breaking residues, positively charged amino acids in the TMD or in the TMD-flanking region could also destabilize the TMD of RIP substrates, as previously reported for rhomboid and S2P substrates that were enriched for the positively charged amino acids arginine (R) and lysine (K) in the TMD-neighboring region (Fleig et al., 2012; Greenblatt et al., 2012; Liu et al., 2020). Moreover, R and K mutation in the Transitional ER ATPase p97 TMD decreased the cleavage efficiency by the mammalian Rhomboid-like 4 (RHBL4), whereas insertion of a patch of arginine amino acids in the TMD of non-RHBL4 substrates led to cleavage by RHBL4 (Fleig et al., 2012). Positively charged amino acids were overrepresented in the TMD of the atMB-TFs in comparison to the TMD of *Arabidopsis* membrane proteins (57% versus 30%; *P* value = 78e-5, hypergeometric distribution; Supplementary Table 5). Next, we assessed the overrepresentation of positively charged amino acids in the TMD-surrounding region of the atMB-TFs. However, as the criteria on the required number of positively charged amino acids and their distance from the TMD were not defined, we searched the atMB-TFs for overrepresentation of arginine and lysine within a 5–, 10–, 15–, and 20-amino-acid window flanking the TMD at the TFFD side, relative to the background *Arabidopsis* proteome and to the corresponding TMD-surrounding region for all *Arabidopsis* membrane proteins. Ten atMB-TFs (NTL1, NTL9, NTL2, bZIP28, bZIP17, bZIP49, AT5G63280, AT2G13960, AT2G29660, and AT5G25475) were detected with a significant (adjusted hypergeometric *P* value < 0.05) overrepresentation of positively charged amino acids in at least one of the tested TMD-flanking regions (Table 1 and Supplementary Table 7). The most significant results, based on the lowest hypergeometric *P* values, were obtained with the 15-amino-acid TMD-flanking region and included NTL1, bZIP17, bZIP28, and bZIP49, known or predicted to be regulated by RIP. The overrepresentation of positively charged amino acids observed in the TMD and in the TMD-flanking regions, respectively, was mainly due to the presence of lysine (38% versus 12%; *P* value = 1.79e-6, hypergeometric distribution and Supplementary Table 7, respectively).Moreover, NTL7 and NTL3, predicted to be activated through (a) rhomboid protease(s), contain at least one lysine/arginine in their TMD and bZIP17, bZIP28, and bZIP49, including bZIP17 and bZIP28 known to be cleaved by S2P, all possess one lysine in the TMD and enrichment of lysine/arginine in the TMD-flanking region, stressing the importance for RIP in plants of positively charged amino acids, and specifically, lysine in and close to the TMD.

### Identification of Proteolytic N-Termini of atMB-TFs

Another strategy to identify proteolytic events is the profiling of N-terminal peptides resulting from cellular or *in vivo* proteolysis. We searched the atMB-TFs for *in vivo* proteolytic N-termini from N-terminomics experiments adopted by the PTM Viewer database (Willems et al., 2019). For more details on N-terminomics assays, we refer the reader to Kaushal and Lee (2021). A proteolytic N-terminus was identified for ANAC028 in a 41-amino-acid proximity of the TMD at the side of the TFFD (Figure 2). The N-terminal arginine of ANAC028 is a N-terminus typically to be expected from RIP, for instance from S1P cleavage. The presence of two lysine residues in its TMD further support ANAC028 as a novel candidate for RIP regulation. However, identification of N-termini that are the consequence of proteolytic MB-TF activation is not straightforward. TFs are generally low-abundant proteins and fragments caused by juxta- or intramembrane proteolysis might be difficult to detect by mass spectrometry, because they are highly hydrophobic and short. Indeed, RIP cleavage regions in and around the TMD are enriched for lysine and arginine, the preferred cleavage sites for trypsin, which is the most widely used peptide generator in mass spectrometry.

### Posttranslational Modifications of atMB-TFs

Phosphorylation and glycosylation of the ER luminal part of human bZIPs CREB4 and NRF1 have been shown to be a first trigger for their proteolytic release (Stirling and O’Hare, 2006; Zhang et al., 2014). For NRF1, glycosylation is essential for its topological repartitioning across the ER membrane by the ERAD complex, whereas deglycosylation of the same amino acid in the translocated TFFD part is crucial for its subsequent proteolytic release from the ER-membrane (Zhang et al., 2014). For the plant NTL11 as well, phosphorylation by the phosphatidylinositol 4-kinase 5 is indispensable for its release and relocalization during auxin-regulated cell division (Tang et al., 2016), but the corresponding phosphorylation site and the exact function of this phosphorylation event have not been identified yet. Moreover, NTM1 was found to be stable in the presence of the proteasome inhibitor MG132, indicating it is ubiquitinated and regulated by rapid protein turnover (Kim et al., 2006). By means of publicly available mass spectrometry data from the PTM Viewer (Willems et al., 2019), posttranslational modifications (PTMs), including phosphorylation events, were searched in the atMB-TFs (Table 1). In total, 27 atMB-TFs had at least one phosphorylation site in their protein sequence and it was located in-between the TFFD and TMD for 19 of them. In addition to phosphorylation, other PTMs, such as a K-acetylation (in RLT2) and an N-glycosylation (in AT5G63280) sites were found between the TFFD and the TMD domain. However, for NTL11, none of the mass spectrometry studies revealed a phosphorylation event, indicating that its phosphorylation only occurs under specific conditions that trigger its activation.

## Biological and Cellular Functions of atMB-TFs

Due to the presence of the TMD, MB-TFs remain in a dormant state, until they are either activated by specific environmental or cellular (for example, hormonal) stimuli, or their function is required to control specific plant developmental programs. The number of MB-TFs, and TFs in general, is higher in plants than that of human/animal systems. For instance, in the human proteome, only six MB-TFs have been reported in two TF families, namely bZIP and zinc finger-NF-X1 (Zupicich et al., 2001). This expansion of the (MB-)TF repertoire in plants reflects the need for a tight transcriptional control and prompt responses as a consequence of their sessile lifestyle and the lack of an adaptive immune system (Shiu et al., 2005). Functional studies of MB-TFs are often associated with subcellular dynamics analyses in response to specific intra- and extracellular stimuli, combined with reversed genetics (gain- and loss-of-function) examinations. For the 52 atMB-TFs, we carried out a systematic literature search for subcellular relocalization and/or functional studies and found that 18 atMB-TFs had a function in cellular or environmental stress responses, 11 were involved in plant development, and 6 functioned in both stress and development (Table 2). For 12 of the 22 atMB-TFs, of which the subcellular localization had been studied by fluorescence tagging, an altered localization pattern could be observed under specific conditions or upon triggers corresponding with the MB-TF function based on reverse genetics analysis (Table 1). However, four atMB-TFs, Myc106, bHLH155, SCP, and SPL7, were exclusively detected in the nucleus, implying either that only a TMD-truncated isoform had been produced, or that the TF had been posttranslationally activated under the experimental conditions.

**TABLE 2:** Overview of known functions of atMB-TFs in plant development and stress responses.

Protein name	Function of atMB-TF			References
	Development	Stress	Description	
AIL6	X		Flower development	Han and Krizek, 2016
bHLH115		X	Fe starvation	Kurt et al., 2019
bZIP17	X	X	Unfolded protein response, root elongation and heat stress response	Liu et al., 2008; Sun et al., 2015; Iwata et al., 2017; Gao et al., 2022
bZIP28	X	X	Unfolded protein response and root elongation	Liu et al., 2007; Sun et al., 2013, 2015; Iwata et al., 2017; Kim et al., 2018
bZIP60		X	Unfolded protein response and heat stress response	Deng et al., 2011; Nagashima et al., 2011; Singh et al., 2021
CAMTA1		X	Drought stress response	Pandey et al., 2013
CAMTA5	X	X	Calcium- dependent root development and drought stress response	Iqbal et al., 2022.
HHO5	X		Floral meristem development	Moreau et al., 2016
LHL2	X		Early seed development	Ohashi-Ito et al., 2013
LOL1		X	Programmed cell death during hypersensitive response	Epple et al., 2003
MAMYB	X		Root hair development	Slabaugh, 2011; Slabaugh et al., 2011
NFXL2	X	X	Cuticle biosynthesis and speed of the circadian clock	Johansson et al., 2011; Lisso et al., 2012
NGAL2		X	Seed size and abiotic stress response	Chen et al., 2019
NLP3		X	Nitrogen starvation	Konishi and Yanagisawa, 2013; Tian et al., 2017
NOK	X		Petal morphogenesis and flowering	Baumann et al., 2007; Hong et al., 2021
NTL1	X	X	Mitochondrial retrograde signaling and seed dormancy	De Clercq et al., 2013; Jurdak et al., 2021
NTL3		X	Mitochondrial retrograde signaling and dark-induced senescence	Broda et al., 2021
NTL4		X	Proteasome activity, ROS damage, senescence and ABA-dependent programmed cell death, drought stress and heat stress	Lee et al., 2012, 2014; [Bibr B107][Bibr B34];
NTL5		X	ABA signaling	Li et al., 2014
NTL6		X	Unfolded protein response	Seo et al., 2010; Yang et al., 2014a
NTL7	X	X	Mitochondrial retrograde signaling, drought stress, flooding and (mitochondrial) unfolded protein response, and (dark-induced) senescence.	Ng et al., 2013; Van Aken et al., 2016; Meng et al., 2019, 2020; Kacprzak et al., 2020; Broda et al., 2021
NTL8	X	X	Abiotic stresses-induced flowering and trichome development	Kim et al., 2007; Tian et al., 2017
NTL9		X	Calcium-dependent programmed cell death during biotic stress responses	Yoon et al., 2008; Block et al., 2014;
NTL11		X	Proteasome activity during abiotic stress	Morishita et al., 2009; Yabuta et al., 2011; Gladman et al., 2016; Tang et al., 2016
NTL13		X	ER-stress induced programmed cell death	Yang et al., 2014b
NTM1		X	Cytokinin-mediated cell division	Kim and Park, 2007
OBP3		X	Light signaling from phytochrome and cryptochrome	Ward et al., 2005
RLT2	X		Phaseolin (major seed storage protein)production	Sundaram et al., 2013
SCP	X		Asymmetric cell division of the gametophyte during pollen development	Chen and McCormick, 1996; Oh et al., 2010; Kim et al., 2015
SPL1		X	Heat-induced inflorescence	Chao et al., 2017
SPL7		X	Cu and Fe starvation response	Bernal et al., 2012; Ramamurthy et al., 2018
SPL12		X	Heat-induced inflorescence	Chao et al., 2017
SRS8	X		Gynoecium development	Kuusk et al., 2006
WIP4	X		Embryogenesis	Listiawan et al., 2015

Six atMB-TFs (NTL6, NTL7, NTL13, bZIP17, bZIP28, and bZIP60) play a role during ER stress by regulating the expression of unfolded protein-responsive genes (Liu et al., 2008; Tajima et al., 2008; Nagashima et al., 2011; Yang et al., 2014a,b; Fuchs et al., 2022; Table 2). Also in rice (*Oryza sativa*) and maize (*Zea mays*), orthologs of bZIP17/28/60 were discovered in ER stress regulation (Hayashi et al., 2012; Li et al., 2012; Takahashi et al., 2012; Yang et al., 2013, 2022; Table 3). The involvement of membrane-bound bZIPs in the UPR is well studied in different eukaryotes and the activation mechanisms are largely conserved. Transcripts of the plant bZIP60, mammalian XBP1, and yeast HAC1 are alternatively spliced by IRE1 isoforms that are activated upon ER stress (for instance, tunicamycin treatment) by oligomerization and autophosphorylation. Consequently, the bZIP mRNAs are alternatively spliced, with a premature stop codon to exclude the TMD as a result (Yoshida et al., 2001; Calfon et al., 2002; Nagashima et al., 2011; Jäger et al., 2012; Diwan et al., 2021). Moreover, for the plant bZIP17 and bZIP28 and the mammalian SREBPs/ATF6, the accumulation of unfolded proteins is sensed in the luminal TF part and results in the translocation of the TFs from the ER to the Golgi, where they are released by the S2P metalloprotease (Ye et al., 2000a,b; Stirling and O’Hare, 2006; Liu et al., 2008; Tajima et al., 2008; Iwata et al., 2017). In mammalian systems, bZIP MB-TFs are activated through phosphorylation (SREBPs) or glycosylation (ATF6) of their luminal part upon unfolded protein accumulation (Hong et al., 2004; Stirling and O’Hare, 2006; Bujalka et al., 2013), but PTMs of their plant counterparts remain to be discovered.

**TABLE 3:** Overview of MB-TFs functions in crop development and stress responses.

Crop	Protein name	TF family	*A. thaliana* ortholog	Function	References
*Brassica napus*	BnaNAC60	NAC	AtNTL5	Programmed cell death and age-triggered leaf senescence	Yan et al., 2021a
	BnaNTL1	NAC	AtNTL7	Leaf senescence	Yan et al., 2021b
*Glycine max*	GmbHLHm1	bHLH		Nodule development, NH^+^ transport	Chiasson et al., 2014
	GmNTL1	NAC	AtNTL1	H_2_O_2_ sensitivity	Li et al., 2016
	GmNTL1 GmNTL4 GmNTL10	NAC		Al toxicity response	Lin et al., 2021
*Lactuca sativa*	LsNAC069	NAC	AtNTL1/3/7	Downy mildew resistance	Meisrimler et al., 2019
*Nicotiana benthamiana*	NbNAC089	NAC	AtNTL14	Virus resistance	Li et al., 2018
	NbNTP1	NAC	AtNTL6	*Phytophthora* resistance	McLellan et al., 2013
	NbNTP2	NAC	AtNTL1/3/7	*Phytophthora* resistance	McLellan et al., 2013
*Oryza sativa*	OsbZIP39	bZIP	AtbZIP28	ER stress response	Takahashi et al., 2012
	OsbZIP50	bZIP	AtbZIP60	ER stress response	Hayashi et al., 2012
	OsbZIP60	bZIP		ER stress response and grain chalkiness	Yang et al., 2022
	OsMADS18	AGL		Seed germination, tiller development and ABA response	Yin et al., 2019
	OsNTL3	NAC		Thermotolerance, ER stress and unfolded protein response	Liu et al., 2020
	OsNTL5	NAC	AtNTL4	Flower development	Guo et al., 2018
*Raphanus raphanistrum*	RsNAC013	NAC	AtNTL1	Oxidative stress response, programmed cell death and pithiness	Hoang et al., 2022
*Solanum lycopersicum*	SlNACMTF2	NAC		Drought and heat stress	Bhattacharjee et al., 2017
	SlNACMTF3 SlNACMTF11	NAC		Viral infection response	Bhattacharjee et al., 2017
	SlNACMTF8	NAC		Drought stress	Bhattacharjee et al., 2017
	SlSRN1	NAC	AtNTL1/3/7	Pathogen resistance	Liu et al., 2014
*Solanum tuberosum*	StNTP1	NAC	AtNTL6	*Phytophthora* resistance	McLellan et al., 2013
	StNTP2	NAC	AtNTL1/3/7	*Phytophthora* resistance	McLellan et al., 2013
*Triticum aestivum*	TaNAC8	NAC		Abiotic stress response and fungal pathogen (Rust) resistance	Xia et al., 2010
	TaNTL1	NAC	ZmNTL1 OsNTL3	Drought resistance and ABA response	Sun et al., 2022
*Zea mays*	ZmbZip17	bZIP	AtbZIP17	ER quality control and ABA signaling	Yang et al., 2013
	ZmbZIP53	bZIP		Gibberellin-regulated germination and plant growth	Lv et al., 2021
	ZmbZIP60	bZIP	AtbZIP60	ER and heat stress response	Li et al., 2012, 2020
	ZmNTL1	NAC		H_2_O_2_ sensitivity	Wang et al., 2016
	ZmNTL2	NAC	AtNTL4/11	H_2_O_2_ sensitivity	Wang et al., 2016
	ZmNTL5	NAC		H_2_O_2_ sensitivity	Wang et al., 2016

Besides the conserved function of MB-bZIPs in UPR in eukaryotes, the plant UPR is also regulated by ER membrane-bound NAC TFs. A nuclear isoform of NTL6 was detected during ER stress, after tunicamycin treatment, as well as during different biotic and abiotic stresses and treatment with the abiotic stress hormone ABA, and resulted in the regulation of UPR, pathogenesis-regulated, and cold-responsive genes (Seo and Park, 2010; Yang et al., 2014a). However, further research is still needed on the exact activation mechanisms and it remains to be assessed whether NTL6 is activated by the accumulation of un- or misfolded proteins in the ER, resulting from excessive demands on the protein folding machinery during environmental stresses (Yang et al., 2014a). NTL13 regulates ER stress-induced programmed cell death in response to ER overreduction by the reducing agent dithiothreitol (DTT) and, in contrast to NTL6, is seemingly not directly involved in UPR, but is responsive to lipid composition changes in the plasma membrane caused by ER dysfunction (Yang et al., 2014b). Interestingly, NTL13 also controls programmed cell death in response to DTT overreduction of the chloroplasts by repressing stromal ascorbate peroxidase-encoding genes (Klein et al., 2012). Moreover, NTL7, well-characterized for its function in mitochondrial stress responses, also plays a role during DTT-induced ER stress by boosting mitochondrial respiration to enable oxidization of excess reducing equivalents from the ER (Fuchs et al., 2022). Whether these membrane NAC and bZIP TFs have distinct and/or overlapping functions in UPR and which are the precise mechanisms of ER stress sensing and their consequent activation are still not understood.

Regulation of the proteasome activity is another function that is mediated by MB-TFs. NTL4 and NTL11 control the expression of proteasome stress regulon-encoding genes, a set of genes discovered co-expressed and essential during short and long proteotoxic stresses provoked by the proteasome inhibitors MG132 and bortezomib. Moreover, their function had been shown to be essential during proteotoxic stress and during heat, drought and high light stress that also impair the plant’s ability to recycle polyubiquitinated proteins (Morishita et al., 2009; Yabuta et al., 2011; Lee et al., 2012, 2014; Shih et al., 2014; Gladman et al., 2016). Also human NRF1 functions in maintaining proteostasis by coordinating the expression of all proteasome subunit genes during proteotoxic stress and NRF1 itself is attenuated by the proteasome as its protein levels were stabilized by MG132 treatment (Sha and Goldberg, 2014). Similarly, for *Arabidopsis* NTL4 and NTL6, rapid protein turnover by the proteasome had been shown (Lee et al., 2012; Gladman et al., 2016).

In total, 11 atMB-TFs have, based on gain- and/or loss-of-function analyses, a proven function in the plant’s responses to environmental stresses, including heat and cold stress (NTL4, NTL6, NTL11, SPL1, SPL12, bZIP17, and bZIP60), drought and salt stress (NTL4, NTL7, NTL8 and NTL9), high light (NTL11), flooding (NTL7) and pathogen attack (LOL1, NTL1, NTL6, and NTL9) (Epple et al., 2003; Kim et al., 2007; Yoon et al., 2008; Morishita et al., 2009; Seo et al., 2010; Deng et al., 2011; Yabuta et al., 2011; Lee et al., 2012, 2014; De Clercq et al., 2013; Ng et al., 2013; Block et al., 2014; Chao et al., 2017; Meng et al., 2020; Singh et al., 2021; Gao et al., 2022; Table 2). Also in non-model species there are multiple examples of stress phenotypes from altered expression of MB-TFs, namely during heat stress (*Oryza sativa* [Os]NTL3 and *Solanum lycopersicum* NAC MEMBRANE-BOUND TRANSCRIPTION FACTOR 2 [SlNACMTF2]), drought (SlNACMTF2, SlNACMTF8, *Triticum aestivum* [Ta]NTL1), Al toxicity (*Glycine max* [Gm]NTL1/4/10) and biotic stress (*Lactuca sativa* LaNAC069, *Nicotiana benthamiana* [Nb]NAC089, NAC TARGETED BY *PHYTOPHTHORA* 1 [NbNTP1] and NbNTP2, SlNACMTF3, SlNACMTF11 and STRESS-RELATED NAC1 [SlSRN1], *Solanum tuberosum* [St]NTP1 and StNTP2, TaNAC8) (McLellan et al., 2013; Xia et al., 2010; Liu et al., 2014, 2020; Bhattacharjee et al., 2017; Li et al., 2018; Meisrimler et al., 2019; Lin et al., 2021; Sun et al., 2022; Table 3). Pre-existing dormant TFs provide an efficient way of gene regulation and enable prompt responses to environmental changes that are necessary for stress adaptation and survival. As several MB-TFs have been reported to mediate reactions to multiple stresses, not surprisingly, several MB-TFs (NTL1, NTL3, NTL4, NTL7, and NTL11) mediate responses to increased cellular ROS production, a common factor under various abiotic and biotic stress conditions (Table 2; Morishita et al., 2009; Lee et al., 2012; De Clercq et al., 2013; Ng et al., 2013). ROS are not just toxic molecules, but also act as secondary messengers under various stress conditions, similarly to calcium. NTL9 is regulated by calcium through binding to calmodulin and plays a role in the regulation of calcium-dependent programmed cell death during effector-triggered immunity (ETI) in addition to its role in osmotic stress-induced leaf senescence (Yoon et al., 2008; Block et al., 2014). Its function during the ETI-induced hypersensitive response is impaired by the *Pseudomonas syringae* pathogenicity-dependent outer protein D1 (HopD1) effector, a strong repressor of ETI. Interaction of HopD1 with NTL9 inhibits the calcium-induced translocation of NTL9 from the ER to the nucleus and the ETI response induction (Block et al., 2014). This finding, i.e., that the NTL9 regulation is affected at the posttranslational level by binding an effector protein, indicates that its activation is most probably regulated posttranslationally through proteolysis. Similarly to the *Arabidopsis* NTL9, the lettuce LsNAC069, an ortholog of *Arabidopsis* NTL1, NTL3, and NTL7, had been shown to be targeted by effectors (i.e., from downy mildew *Bremia lactucae*) that abolished its relocalization to the nucleus (Meisrimler et al., 2019). NTL1, NTL3, and NTL7 have mainly been studied for their function in mitochondrial retrograde signaling, in which stressed or dysfunctional mitochondrial status (for instance, by treatment with the mitochondrial complex III inhibitor antimycin A) is communicated to the nucleus to induce adaptation responses for the maintenance of the mitochondrial as well as the whole cellular homeostasis (De Clercq et al., 2013; Ng et al., 2013; Van Aken et al., 2016; Broda et al., 2021). Since their discovery as regulators of mitochondria-to-nucleus communication pathways, NTL1, NTL3, and NTL7 were studied in the context of various cellular and environmental stress responses, including the mitochondrial and ER unfolded protein responses (Kacprzak et al., 2020; Fuchs et al., 2022), drought (Van Aken et al., 2016), and flooding stress (Meng et al., 2020). NTL7 is constitutively produced and acts as a ‘master switch’ that regulates the expression of *NTL1*, *NTL3* and *NTL4* as downstream targets (Van Aken et al., 2016; Broda et al., 2021). Mutant analyses have revealed that the fine-tuned expression of these NTLs is necessary to sustain a normal development, because misregulation of their expression resulted in deficient seed dormancy breaking (NTL3) (Jurdak et al., 2021), accelerated dark-induced senescence (NTL3 and NTL7) (Meng et al., 2019; Broda et al., 2021), and growth retardation and altered leaf development due to decreased cell size and viability (NTL7) (Meng et al., 2019).

Among the MB-TFs with a described function in plant development, the majority plays a role in the control of developmental stages from flower development to seed generation (SCP, bHLH155, WOUND-INDUCED POLYPEPTIDE 4, AINTEGUMENTA-LIKE 6, HRS1 HOMOLOG, NTL4, NTL8, NTL11, SHI-RELATED SEQUENCE 8, RNASE THREE-LIKE PROTEIN 2, and NF-X LIKE 2), and in root hair development or in root elongation (MAMYB, bZIP17, bZIP28, and FRF3) (Chen and McCormick, 1996; Kuusk et al., 2006; Kim et al., 2007; Morishita et al., 2009; Oh et al., 2010; Johansson et al., 2011; Lisso et al., 2012; Sundaram et al., 2013; Shih et al., 2014; Kim et al., 2015; Listiawan et al., 2015; Han and Krizek, 2016; Moreau et al., 2016; Tian et al., 2017; Kurt et al., 2019; Table 2). Also in non-model organisms several MB-TFs are involved in development, including flower development (OsNTL5), seed germination (OsMINICHROMOSOME MAINTENANCE 1/AGAMOUS/DEFICIENS/SERUM RESPONSE FACTOR 18 and ZmbZIP53), aging (BnaNAC60 and BnaNTL1) and nodule formation GmbHLH membrane 1) (Chiasson et al., 2014; Guo et al., 2018; Yin et al., 2019; Lv et al., 2021; Yan et al., 2021a,b; Table 3). The ER-to-nucleus mobilization of MAMYB specifically takes place in root epidermal cells adjacent to the sites of lateral root initiation and loss-of-function mutation results in absence of lateral roots (Slabaugh et al., 2011). Another well-studied MB-TF is SCP that plays a role in the regulation of cell division during pollen development. SCP has been detected in the nucleus, specifically during early and polarized microspore stages, and functions in the control of asymmetric cell division of the gametophyte during pollen development (Chen and McCormick, 1996; Oh et al., 2010; Kim et al., 2015). Although these TFs seem to be activated in specific cell types, in which their activity is required to mediate developmental programming, their specific activation mechanism in a cellular and developmental stage specific manner, has not been elucidated yet. Developmental programming is known to be also controlled by environmental stimuli. Flower development and seed germination are triggered by changes in photoperiod and temperature (Baskin and Baskin, 2004; Song et al., 2013), whereas lateral root growth that is regulated by hormones, such as auxin, is also responsive to environmental stimuli, such as nutrient deficiency and soil water content (Banda et al., 2019). For instance, NTL4 and NTL11 regulate jasmonic acid (JA)- and ABA-dependent and high-light-induced florescence, respectively (Yabuta et al., 2011; Shih et al., 2014; Tang et al., 2016). However, how various intra-, inter- and extracellular signals contribute to and are intersected for the MB-TF activation is not understood.

**FIGURE 4:**
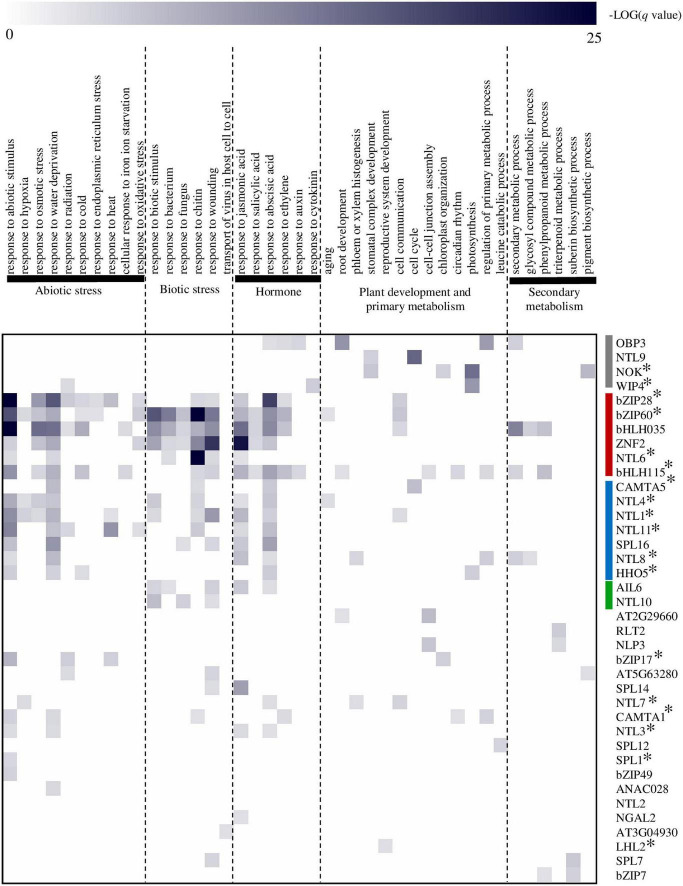
Prediction of atMB-TF functions by means of a regulatory network-based approach. Functional terms were obtained per TF by Gene Ontology (GO) Biological Process (BP) enrichment analysis of their respective target genes (De Clercq et al., 2021). MB-TFs were hierarchically clustered (average linkage) based on their functional categories using Genesis software version 1.6 (Sturn et al., 2002). atMB-TFs that regulate a broad range of abiotic and biotic stress responses are in indicated in red; those involved mainly in responses to water deficit and ABA, in blue; mostly involved in biotic stress responses, in green; and those in specific cellular and/or developmental processes, in gray. A selection of GO-BP terms are displayed for the heatmap presentation. For the complete list of enriched GO-BP terms per atMB-TF, see Supplementary Table 3. *Indicates atMB-TFs with correctly predicted function(s).

To discover novel functions for yet uncharacterized atMB-TFs, we used a transcriptional regulatory network approach for the identification of TF functions based on their target genes (De Clercq et al., 2021). This method has a high predictive power to correctly infer functions for both functionally characterized and novel TFs involved in various biological processes. For 38 atMB-TFs, at least one enriched gene ontology (GO) biological process (BP) term was obtained, with in total 291 GO-BP terms used for hierarchical clustering of the MB-TFs according to their predicted function(s) (De Clercq et al., 2021; Figure 4 and Supplementary Table 3). We could distinguish atMB-TFs that regulate (i) a broad range of abiotic and biotic stress responses, (ii) mainly responses to water deficit and ABA, (iii) mostly biotic stress responses, and (iv) specific cellular and/or developmental processes (Figure 4). This network-based identification could assign one or more of the known functions for 18 of the 35 functionally characterized atMB-TFs (Table 2), whereas for the remaining part, no enriched GO terms were found (9/35) or novel functions were assigned (9/35). MB-TFs involved in the same or similar biological processes cluster together in our meta-analysis and are, among other functions, enriched for unfolded protein response (i.e., bZIP28 and bZIP60; group i) and water deprivation functions (i.e., NTL4, NTL8, and HHO that control drought stress-triggered flowering; group ii) (Kim et al., 2007; Shih et al., 2014; Moreau et al., 2016). For 12 out of the 17 atMB-TFs without known functions, novel roles could be predicted. These yet uncharacterized atMB-TFs are expected to function in stress responses, namely bHLH035 and SPL16 to be involved in a broad range of biotic and abiotic stresses, bZIP49 and NTL2 in abiotic stress responses, and NTL10 and ZFN2 in biotic stress responses. Furthermore, specific functions were assigned to the other atMB-TFs: water deprivation (ANAC028), cell-to-cell transport of viruses (AT3G04930), JA responses (SPL14), regulation of root development and cell junctions (AT2G29960), regulation of pigment biosynthesis (AT5G63280), and regulation of the phenylpropanoid pathway and suberin biosynthesis (bZIP7). However, further experimental studies are needed to validate these functional predictions.

## Conclusion and Further Perspectives

MB-TFs play an important role in the regulation of various cellular processes and unraveling their mode of action provides important insights into the molecular mechanisms of how plants sense and coordinate intra- and intercellular and environmental signals into appropriate responses. However, our knowledge on the activation mechanisms of MB-TFs in plants is limited to that of bZIP17 and bZIP28, and bZIP60 that are regulated through RIP and alternative spicing, respectively, during the unfolded protein response (Liu et al., 2008; Tajima et al., 2008; Nagashima et al., 2011). Both activation mechanisms are highly conserved in multicellular life (Sun et al., 2015; Diwan et al., 2021). Experimental indication for proteolytic activation of MB-TFs is often based on N-terminomics or pharmacological methods with certain protease inhibitors. Both approaches have their limitations, because peptides derived from RIP are often difficult to detect by mass spectrometry and pharmacological systems are restricted to currently available bacterial and mammalian small molecule inhibitors, of which action mode in plants is often not well known. Moreover, chemical inhibitor studies rely on previous knowledge of a readout (specific trigger and timing) of the MB-TFs activity. In plants, this approach has been applied only to NTL7 and NTM1, by means of a broad-spectrum serine/rhomboid and calpain protease inhibitor, respectively (Kim et al., 2006; Ng et al., 2013). Furthermore, *in silico*-based prediction of proteolytic activation is unsatisfactory because of the short and degenerate nature of known protease recognition sequences. Structural features within or adjacent to the TMD, such as helix-breaking motifs and positively charged amino acids, rather than sequence motifs are seemingly more relevant to predict cleavage events (Ye, 2020). Here, we identified 11 and 30 atMB-TFs with at least one helix-breaking motif and positively charged amino acid inside the TMD, respectively, among which MAMYB, SCP, NTL3, NTL7, bZIP17, bZIP49, bHLH035, and AT5G25475 have both features, hinting at activation through RIP.

For the subsequent identification of the responsible protease, a forward genetics approach is not straightforward, because the protease activity readout is often indirect and based on the expression of downstream target genes. In the case of bZIP28, the downstream target gene analysis in the *s1p* mutant identified S1P as the responsible protease, although its cleavage was not affected in this mutant (Iwata et al., 2017). A genetics approach is also hampered by potential redundancy between proteases that can cleave one MB-TF (Iwata et al., 2017). Therefore, a CRISPR-Cas library screen targeting multiple proteases simultaneously, in random combinations, may be a useful alternative (Callies et al., 2019). A more direct method for the detection of the responsible protease is chemical genomics using a chemical protease inhibitor as a bait. State-of-the-art techniques to discover protein targets of small molecules are Target Identification by Chromatographic Co-Elution (TICC) (Chan et al., 2012), Drug Affinity Responsive Target Stability (DARTS) (Lomenick et al., 2011), and Activity-Based Protein Profiling (ABPP) (Sieber et al., 2004), but require specific research resources and expertise. Traditional affinity purification experiments using the MB-TF substrate as a bait are rather unlikely to pull down the responsible protease, because the protease–substrate interactions are weak and transient and, thus, will probably get lost during the purification steps. On the contrary, proximity-dependent labeling techniques circumvent this problem by tagging all interacting/neighboring proteins before pull-down and purification and they are also ideal to detect protein interactions in a membranous environment (Arora et al., 2020). A combination of N-terminomics, chemical biology, and proximity labeling-based interactomics is promising to elucidate the activation mechanism of plant MB-TFs.

Manipulation of the MB-TFs levels by gene knockout or overexpression in *Arabidopsis* and non-model species has been shown to impact the plant’s tolerance or resistance to stresses, but as a drawback, often perturbs growth and development, making this approach unfavorable for agricultural applications. In contrast, expression of a constitutively active isoform of the endogenous MB-TF, for example, by CRISPR-Cas-mediated gene editing of the TMD, provides a valuable alternative. This was shown for *Arabidopsis* NTL7 for which overexpression of the full length protein resulted in growth and developmental retardation in addition to increased oxidative stress tolerance, whereas exclusion of the TMD from the endogenously expressed transcript induced stress tolerance without affecting the growth and development (Ng et al., 2013; Meng et al., 2019; Broda et al., 2021). Moreover, understanding of the cleavage mechanisms offers possibilities to fine tune the proteolytic activation by the native or by alternative proteases. For example, mutation of the cleavage site or addition of positively charged amino acids in or around the TMD was shown to promote cleavage or enable cleavage by alternative proteases in mammals (Beel and Sanders, 2008; Fleig et al., 2012; Greenblatt et al., 2012; Liu et al., 2020; Silber et al., 2020; Spitz et al., 2020; Ye, 2020). Different MB-TFs have already been studied in agricultural important crops showing their involvement in responses to drought stress and infection by viral and fungal pathogens and in various developmental traits such as flowering time and seed germination (Xia et al., 2010; Liu et al., 2014; Bhattacharjee et al., 2017; Li et al., 2018; Guo et al., 2018; Yin et al., 2019; Table 3). A thorough understanding of their activation mechanisms will offer perspectives to fine tune their activity with respect to applications for crop improvement.

## Author Contributions

IDC conceived this review manuscript. JDB summarized and analyzed the literature and performed the *in silico* analyses. IDC and FVB contributed to the discussion and writing of the manuscript. JDB and IDC wrote the manuscript. All authors contributed to the article and approved the submitted version.

## Conflict of Interest

The authors declare that the research was conducted in the absence of any commercial or financial relationships that could be construed as a potential conflict of interest.

## Publisher’s Note

All claims expressed in this article are solely those of the authors and do not necessarily represent those of their affiliated organizations, or those of the publisher, the editors and the reviewers. Any product that may be evaluated in this article, or claim that may be made by its manufacturer, is not guaranteed or endorsed by the publisher.
