# Supplementary material for: Proteolytic Activation of Plant Membrane-Bound Transcription Factors
Source: Front Plant Sci. 2022 Jun 14;13:927746. doi: 10.3389/fpls.2022.927746 (PMC9237531; doi:10.3389/fpls.2022.927746)
Supplement: Supplementary file 2 [file Table_7.docx]

**Supplementary Table S7 | Enrichment analysis of positively charged amino acids in different TMD-adjacent regions of the atMB-TFs**

| **name** | **5-amino-acid flanking region** | | | | **10-amino-acid flanking region** | | | | | | **15-amino-acid flanking region** | | | | | | **20-amino-acid flanking region** | | | | | |
| --- | --- | --- | --- | --- | --- | --- | --- | --- | --- | --- | --- | --- | --- | --- | --- | --- | --- | --- | --- | --- | --- | --- |
|  | **Lysine (K)** | | **Positive AA [RK]** | | **Lysine (K)** | | **Arginine (R)** | | **Positive AA [RK]** | | **Lysine (K)** | | **Arginine (R)** | | **Positive AA [RK]** | | **Lysine (K)** | | **Arginine (R)** | | **Positive AA [RK]** | |
|  | **ratio** | **p-value^*^** | **ratio** | **p-value^*^** | **ratio** | **p-value^*^** | **ratio** | **p-value^*^** | **ratio** | **p-value^*^** | **ratio** | **p-value^*^** | **ratio** | **p-value^*^** | **ratio** | **p-value^*^** | **ratio** | **p-value^*^** | **ratio** | **p-value^*^** | **ratio** | **p-value^*^** |
| NTL1 | 4/5 | 3,41E-07 | 4/5 | 3,07E-03 | 5/10 | 3,37E-05 |  |  |  |  | 6/15 | 9,22E-05 |  |  | 7/15 | 8,56E-03 | 7/20 | 1,02E-04 |  |  | 8/20 | 2,05E-02 |
|  |  | **7,33E-05** |  |  |  |  |  |  |  |  |  | **2,03E-04** |  |  |  | **1,79E-02** |  | **2,92E-05** |  |  |  | **1,05E-02** |
| NTL9 |  |  |  |  |  |  | 4/10 | 1,94E-03 | 6/10 | 1,28E-03 |  |  |  |  |  |  |  |  | 5/20 | 4,03E-02 |  |  |
|  |  |  |  |  |  |  |  | **2,00E-02** |  | **1,54E-02** |  |  |  |  |  |  |  |  |  | **2,91E-02** |  |  |
| NTL2 | 3/5 | 3,79E-03 |  |  |  |  |  |  |  |  |  |  |  |  |  |  |  |  |  |  |  |  |
|  |  |  |  |  |  |  |  |  |  |  |  |  |  |  |  |  |  |  |  |  |  |  |
| bZIP28 | 3/5 | 3,79E-03 |  |  | 5/10 | 3,37E-05 |  |  |  |  | 7/15 | 2,77E-07 |  |  | 7/15 | 8,56E-03 | 7/20 | 1,02E-04 |  |  | 8/20 | 2,05E-02 |
|  |  |  |  |  |  | **6,18E-04** |  |  |  |  |  | **8,27E-07** |  |  |  | **1,79E-02** |  | **2,92E-05** |  |  |  | **1,05E-02** |
| bZIP17 |  |  |  |  | 4/10 | 1,23E-02 |  |  |  |  | 7/15 | 2,77E-07 |  |  | 7/15 | 8,56E-03 | 7/20 | 1,02E-04 |  |  |  |  |
|  |  |  |  |  |  |  |  |  |  |  |  | **8,27E-07** |  |  |  | **1,79E-02** |  | **2,92E-05** |  |  |  |  |
| bZIP49 | 3/5 | 3,79E-03 |  |  | 4/10 | 1,23E-02 |  |  |  |  | 6/15 | 9,22E-05 |  |  | 7/15 | 8,56E-03 | 6/20 | 6,42E-03 |  |  |  |  |
|  |  |  |  |  |  |  |  |  |  |  |  | **2,03E-04** |  |  |  | **1,79E-02** |  | **2,61E-03** |  |  |  |  |
| AT5G63280 | 3/5 | 3,79E-03 |  |  |  |  |  |  |  |  |  |  |  |  |  |  |  |  |  |  |  |  |
|  |  |  |  |  |  |  |  |  |  |  |  |  |  |  |  |  |  |  |  |  |  |  |
| AT2G13960 |  |  |  |  |  |  | 4/10 | 2,00E-02 |  |  |  |  |  |  |  |  |  |  |  |  |  |  |
|  |  |  |  |  |  |  |  | **2,00E-02** |  |  |  |  |  |  |  |  |  |  |  |  |  |  |
| AT2G29660 |  |  |  |  |  |  |  |  |  |  | 5/15 | 1,79E-02 | 27% |  | 9/15 | 1,38E-05 | 5/20 |  |  |  | 9/20 | 4,90E-04 |
|  |  |  |  |  |  |  |  |  |  |  |  | **1,79E-02** |  | **2,13E-01** |  | **1,38E-05** |  | **9,94E-02** |  |  |  | **4,90E-04** |
| AT5G25475 |  |  |  |  |  |  |  |  |  |  |  |  |  |  |  |  |  |  | 5/20 | 2,91E-02 |  |  |
|  |  |  |  |  |  |  |  |  |  |  |  |  |  |  |  |  |  |  |  | **2,91E-02** |  |  |

AA, amino acid.

^*^Hypergeometric *P* value corrected for multiple testing using Bonferroni correction with the top value compared to the *Arabidopsis* proteome (retrieved from Uniprot) and the bottom value compared to all *Arabidopsis* membrane proteins as background (obtained via the *Arabidopsis* membrane protein library; Ward, 2001)
