# Supplementary figures and images for: Proteolytic Activation of Plant Membrane-Bound Transcription Factors
Source: Front Plant Sci. 2022 Jun 14;13:927746. doi: 10.3389/fpls.2022.927746 (PMC9237531; doi:10.3389/fpls.2022.927746)

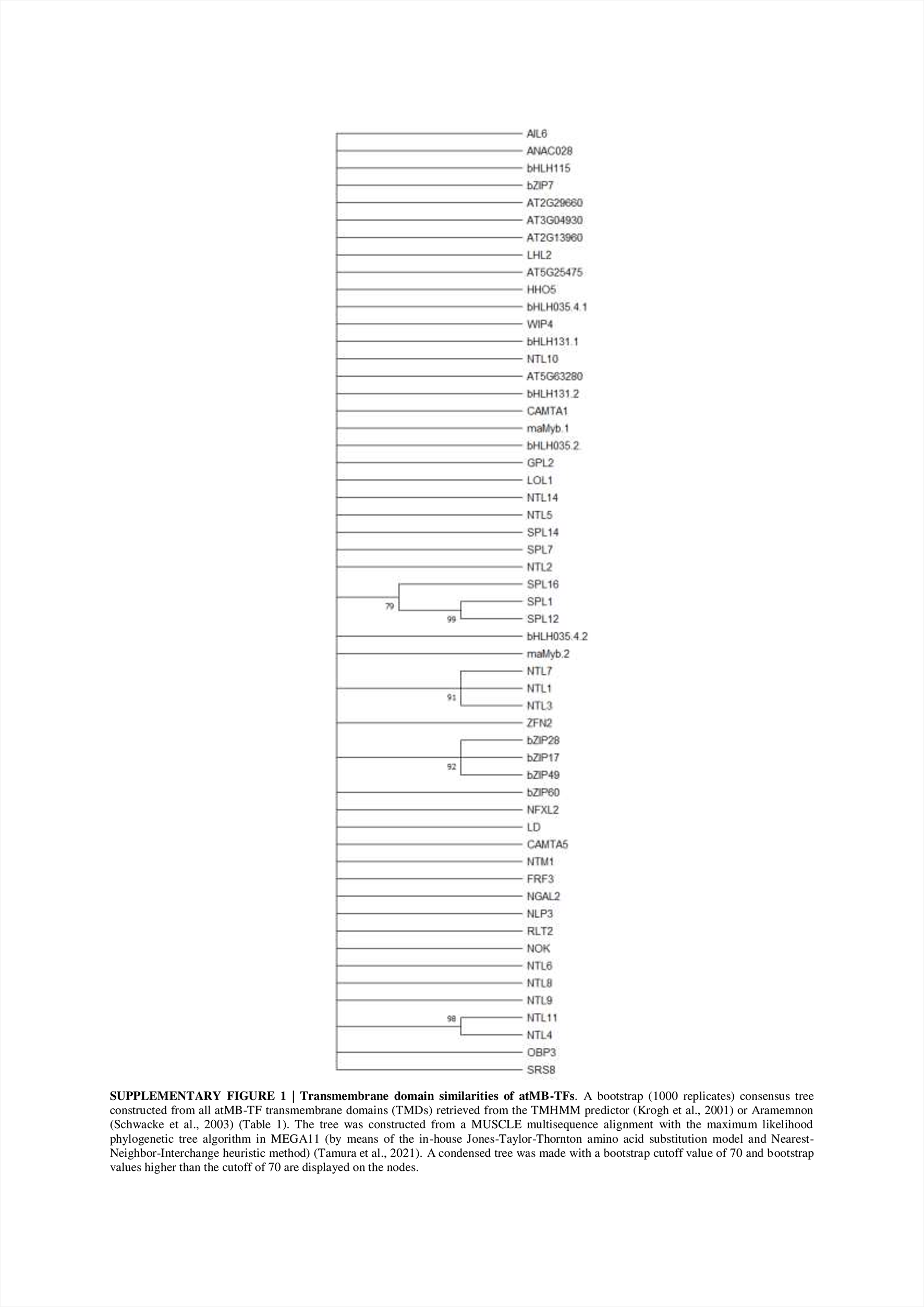

Supplement: Supplementary file 3 [file Image_1.TIFF]
